# Consistency of pacing profile according to performance level in three different editions of the Chicago, London, and Tokyo marathons

**DOI:** 10.1038/s41598-022-14868-6

**Published:** 2022-06-24

**Authors:** Fran Oficial-Casado, Jordi Uriel, Irene Jimenez-Perez, Márcio Fagundes Goethel, Pedro Pérez-Soriano, Jose Ignacio Priego-Quesada

**Affiliations:** 1 Research Group in Sports Biomechanics (GIBD), Department of Physical Education and Sports, Faculty of Physical Activity and Sport Sciences, University of Valencia, C/ Gascó Oliag, 3, 46010 Valencia, Spain; 2 Instituto de Biomecánica (IBV), Universitat Politècnica de València, Valencia, Spain; 3 Research Group in Medical Physics (GIFIME), Department of Physiology, University of Valencia, Valencia, Spain; 4 Centre of Research, Education, Innovation and Intervention in Sport (CIFI2D), Faculty of Sport, University of Porto, Porto, Portugal; 5 Porto Biomechanics Laboratory (LABIOMEP-UP), University of Porto, Porto, Portugal

**Keywords:** Computational biology and bioinformatics, Health care

## Abstract

Running pacing has become a focus of interest over recent years due to its relationship with performance, however, it is still unknown the consistency of each race in different editions. The aim of this study is to analyze the consistency of pacing profile in three consecutive editions of three marathon races. A database of 282,808 runners, compiled from three different races (Chicago, London, and Tokyo Marathon) and three editions (2017, 2018, and 2019) was analyzed. Participants were categorized according to their time performance in the marathon, every 30 min from 2:30 h to sub-6 h. The relative speed of each section for each runner was calculated as a percentage of the average speed for the entire race. The intraclass correlation coefficients (ICC) of relative speed at the different pacing section, taking into account the runner time categories, was excellent over the three marathon editions (ICC > 0.93). The artificial intelligence model showed an accuracy of 86.8% to classify the runners' data in three marathons, suggesting a consistency between editions with identifiable differences between races. In conclusion, although some differences have been observed between editions in certain sections and marathon runner categories, excellent consistency of the pacing profile was observed. The study of pacing profile in a specific marathon can, therefore, be helpful for runners, coaches and marathon organizers for planning the race and improving its organization.

## Introduction

Marathons events are becoming increasingly popular, with a high number of participants (> 30,000 in many cases)^1^, where the main goal for runners is to regulate energy resources to avoid premature fatigue and so finish the race^2,3^. Pacing, considered as the evolution of speed throughout the competition depending on runners' priorities, capabilities, physiological, psychological, energetic, and environmental factors^3–5^, has increased the focus of interest over recent years due to its relationship with performance^2,3,6–9^. A pacing profile can be affected by factors such as age^10–14^, sex^7,9,15–18^, metabolic and cardiovascular factors^19,20^, performance level^8,21–24^, previous experience^2,25^, footwear^26^, collective behavior^27^ or race characteristics^8^. Although more attention has been paid to several factors such as sex or performance level, others like individual race characteristics need further investigation^8^.

In relation to individual race characteristics, Haney and Mercer^28^ analyzed Las Vegas and San Diego marathons without observing any differences between them in terms of pacing profile. Both races had similar slope profiles and they suggested that larger differences in this characteristic could have a greater effect on pacing^28^. A recent study assessed four marathons (Valencia, Chicago, London, and Tokyo marathons) observing differences in their pacing characteristics (e.g., the difference in relative speed between the first and second half of the marathon)^8^, and slope profile and environmental temperature were suggested as possible explanatory factors^8^. Although pacing profile could be different depending on each race, it is important to investigate whether this profile is consistent in different race editions. For example, a consistent pacing profile of races would allow runners to analyze race characteristics prior to the competition and plan pacing during the race, and would enable organizers to analyze pacing profile to take decisions on the organization of the race (where to start and finish, race circuit, starting dynamics, etc.).

The aim of this study, therefore, was to analyze the consistency of pacing profile in three consecutive editions of three marathon competitions. It was hypothesized that the consistency of the pacing profile would be high, since, in the races analyzed, there had been no changes made to the route, and any reduction in consistency would be observed if the environmental conditions differed between editions.

## Materials and methods

### Study design

One possibility to carry out this study was to measure the reproducibility from the data of runners of different categories who participated in three editions of the same marathon. However, this type of design has significant limitations, such as the difficulty of getting enough sample in several different marathons and the fact that the runners’ performance cannot be guaranteed to be the same for three years. For this reason, it was decided to study consistency using three different statistical techniques from an extensive database: intraclass correlation study, analysis of differences through ANOVAs, and artificial intelligence.

This manuscript is a continuation of a previous publication^8^. In this last publication, the database was based on only one edition of four marathons to assess if different pacing profiles were observed. The analysis of this data evocated a new question about the assessment of the consistency of the marathons. Therefore, in the current study, three different editions of three marathons were assessed.

### Database

This study was approved by the Ethical Committee for Research in Humans of the University of Valencia (ref. H1544598666277) and followed the relevant European regulations for data protection and is in accordance with the Declaration of Helsinki. An automatic web scraping application developed in MATLAB (Mathworks Inc., Natick, USA), was employed. This application is designed to obtain the runners' times at different kilometer points of a marathon: 5, 10, 15, 21 km, 25, 30, 35, 40 and 42.2 km. All data were obtained and analyzed anonymously from the official and public websites of three editions (2017, 2018, and 2019) and three marathons' races: Chicago Marathon (USA; https://chicago-history.r.mikatiming.com/2019/), London Marathon (UK; https://www.tcslondonmarathon.com/results/race-results), and Tokyo Marathon (Japan; https://www.marathon.tokyo/en/about/past/). These marathons were selected because were gold label races, with an important number of participants, and with no alteration of their route in the last three editions. Inclusion criteria were to have full pacing data registered on the website and to finish the marathon in less than 6 h. From the database obtained, outliers were identified by the intersection of different outlier detection methods defined by the package "OutlierDetection" in RStudio (version 1.2.5033) and removed from the database. The sample size of the total database was, therefore, 282,808 runners. Table 1 shows the characteristics of the marathons assessed.

**Table 1:** Characteristics of the marathons assessed.

Race	Chicago			London			Tokyo		
Edition (year)	2017	2018	2019	2017	2018	2019	2017	2018	2019
Date (day/month)	08/10	07/10	13/10	23/04	22/04	28/04	26/02	25/02	03/03
Number of participants assessed	36,792	38,619	39,503	33,522	32,127	35,870	18,756	26,564	21,055
Mean environmental temperature (°C)*^1^	23	16	11	12	22	12	12	6	5
Mean environmental humidity (%)*^1^	74	89	60	65	75	69	31	45	76
Elevation gain (m)*^2^	11			37			22		
Elevation loss (m)*^2^	21			60			48		

*^1^Environmental conditions were obtained for Tokyo from the official website of the marathon, and for London and Chicago from the website https://www.timeanddate.com. *^2^Elevation gain and loss were obtained from the data of a runner that performed all the marathons (Garmin 920XT, Garmin Ltd, Switzerland).

### Data analysis

Participants were categorized according to their finish time in the marathon: sub-2:30 (n = 812), sub-3:00 (between 2:30 and 2:59 h, n = 12,503), sub-3:30 (between 3:00 and 3:29 h, n = 32,038), sub-4:00 (between 3:30 and 3:59 h, n = 54,727), sub-4:30 (between 4:00 and 4:29 h, n = 56,229), sub-5:00 (between 4:30 and 4:59 h, n = 55,436), sub-5:30 (between 5:00 and 5:29 h, n = 41,306) and sub-6:00 (between 5:29 and 5:59 h, n = 29,757). Full marathon average speed and the average speed of each section were calculated individually. The relative speed of each section for every runner was then calculated as a percentage of the average speed of whole race^6^. Other pacing variables were calculated^7,8^: Pacing range (difference between the maximum and the minimum relative speed obtained in the sections), DifHalf (difference in relative speed between the first half of the marathon and the second half), and ΔRelative speed (difference in relative speed between the km 10 section and km 40 section).

### Statistical analysis

Statistical analysis was performed using RStudio software (version 1.2.5033). To assess the consistency of the pacing profile of the different marathons, the intraclass correlation coefficient (ICC), differences between editions using ANOVAs, and the artificial intelligence method was analyzed. The ICC from the model, based on an average measurement, absolute-agreement, and 2-way random-effects model ("2,*k*")^29^, was calculated between the three years at each of the marathons and runner time categories for the evolution of the relative speed throughout the race. To be able to compare these values with the differences between the marathons, the same ICC analysis was performed, but between the marathons. ICC values were also calculated between the three years for each pacing variable: Pacing range, DifHalf, and ΔRelative speed. The following classification of ICC values was used^30^: values 1.00 to 0.81 (excellent reproducibility), 0.80 to 0.61 (very good), 0.60 to 0.41 (good), 0.40 to 0.21 (reasonable) and, from 0.20 to 0.00 (poor). This type of ICC model was used considering that mean values for each edition were compared and no single runner's data that were involved in the three editions.

To analyze the differences between marathon editions, parametric tests were used due to the large sample sizes in all the groups assessed^31^. Repeated measures ANOVAs, with one within-subject factor (pacing section) and one inter-subject factor (edition), were applied for the relative speed for each marathon and runner time categories. Bonferroni post-hoc test and Cohen's effect sizes (ES)^32^ were applied for pair-comparisons. One-way ANOVAs were performed to assess the differences between marathon editions using pacing profile variables (Pacing range, DifHalf, and ΔRelative speed). The significance level was set at *p* < 0.05 and ES > 0.8 (large effect size) to ascertain a non-overlap in mean scores greater than 47%^32^. Data are reported as mean ± standard deviation in the figures with 95% confidence intervals of the differences (CI95%) in the text.

**Figure 1:**
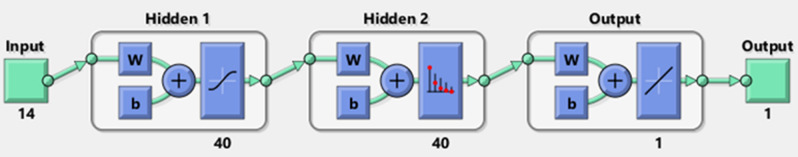
Artificial neural network structure.

If marathons present a consistency between editions but differ from other marathons, artificial intelligence would identify the marathon assessing pacing profile data. Therefore, a feedforward-type network structure of an artificial neural network (ANN) (Fig. 1) with 2 hidden layers was constructed, containing 40 neurons in each layer. The transfer function used in the first layer was the tansigmoidal and in the second softmax. Learning took place by reverse propagation with Bayesian regularization. The data included in the ANN as inputs were the average speed at each section, the runner's category, and the three profile variables assessed. The dataset was randomly divided into proportions of 70% for training and 30% for testing. The training performance was demonstrated through the mean square error value, which reached its minimum value of 0.132 after 604 epochs. ANN analysis was performed using Matlab (version 2020b, The Math Works Inc., Natick, MA, USA).

## Results

**Figure 2:**
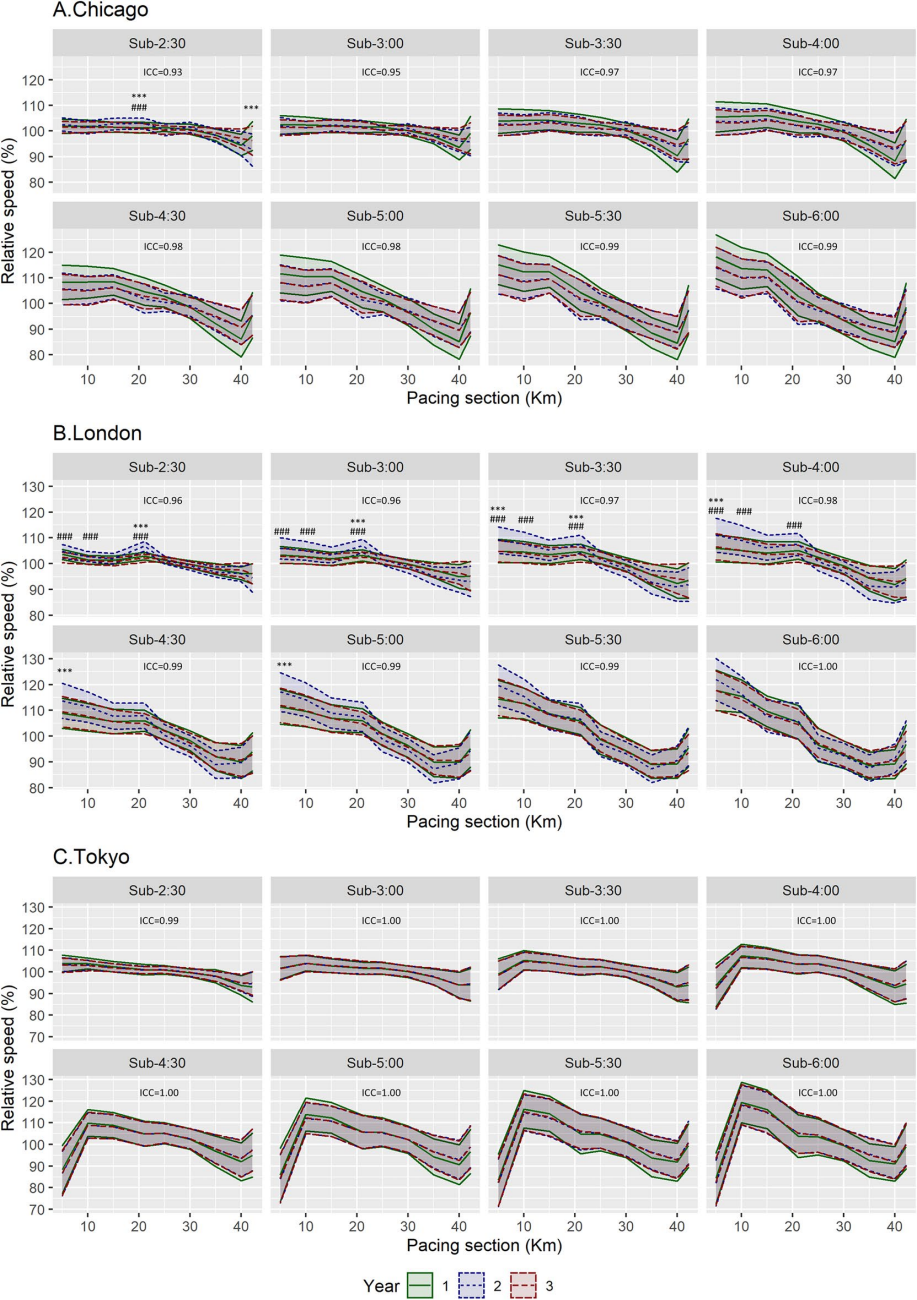
Mean and standard deviation of relative speed (percentage of average speed for the full marathon) at the different pacing sections assessed for the different marathon editions. The intraclass correlation coefficient (ICC) between years is shown for each marathon and runner time category. Differences (*p* < 0.05 and ES > 0.8) between years are shown using symbols (*** *p* < 0.001 and ES > 0.8 between 2017 and 2018; ### *p* < 0.001 and ES > 0.8 between 2018 and 2019; no differences being observed between 2017 and 2019 editions).

The ICC of relative speed at the different pacing sections, taking runner time categories into account, was excellent between the three marathon editions (ICC > 0.93) (Fig. 2). The 2018 Chicago edition presented higher relative speed for sub-2:30 runners at km 21.1 than the other two editions (vs. 2017 CI95% [1.0, 2.3%] *p* < 0.001 and ES = 0.8; vs. 2019 CI95% [1.3, 2.3%] *p* < 0.001 and ES = 0.9), lower relative speed at the end of the race compared with the 2017 edition for sub-2:30 (CI95% [− 3.7, − 7.5%] *p* < 0.001 and ES = 0.9), and lower relative speed at the end of the race compared with the 2019 edition for sub-4:00 runners (CI95% [− 0.7, − 1.1%] *p* < 0.001 and ES = 0.8). Overall, the 2018 London edition presented higher relative speed in the first 21 km than the other two editions for runners from sub-2:30 (e.g. km 21.1: vs. 2017 CI95% [3.0, 4.7%] *p* < 0.001 and ES = 2.4; vs. 2019 CI95% [3.8, 5.4%] *p* < 0.001 and ES = 2.8) to sub-4:00 (e.g. km 5: vs. 2017 CI95% [4.9, 5.3%] *p* < 0.001 and ES = 0.9; vs. 2019 CI95% [4.3, 4.7%] *p* < 0.001 and ES = 0.8), and just at the beginning of the race compared with the 2017 edition for sub-4:30 (CI95% [4.5, 5.0%] *p* < 0.001 and ES = 0.8) and sub-5:00 runners (CI95% [5.5, 6.0%] *p* < 0.001 and ES = 0.8). The Tokyo editions did not differ in any of the runner time categories and pacing sections (*p* > 0.05 and ES > 0.8) and no differences were observed between the 2017 and 2019 editions in any of the marathons assessed (*p* > 0.05 and ES > 0.8).

**Figure 3:**
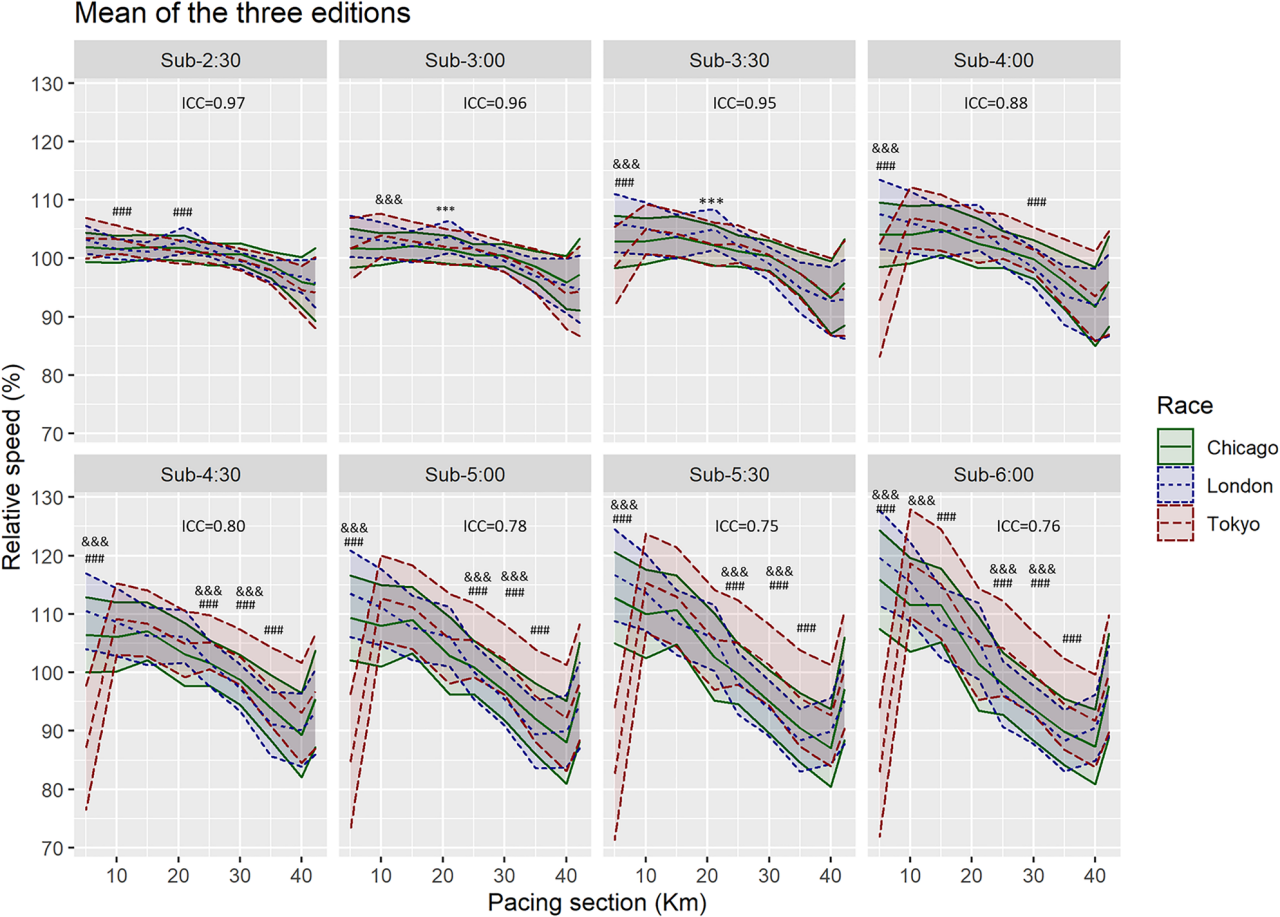
Mean and standard deviation of relative speed (percentage of average speed for the full marathon) at the different pacing sections of the marathons analyzed. The intraclass correlation coefficient (ICC) between marathons is shown for each of the runner time categories. Differences (*p* < 0.05 and ES > 0.8) between marathons are shown using symbols (*** *p* < 0.001 and ES > 0.8 between Chicago and London; &&& *p* < 0.001 and ES > 0.8 between Chicago and Tokyo; ### *p* < 0.001 and ES > 0.8 between London and Tokyo).

Figure 3 shows the differences and ICC values between the three marathons regardless of the annual edition. The figure exemplifies how ICC values were lower when comparing different marathons (ICC 0.75–0.97), especially for the higher runner time categories, than for different editions of the same marathon (Fig. 2; ICC 0.93–1.00).

**Figure 4:**
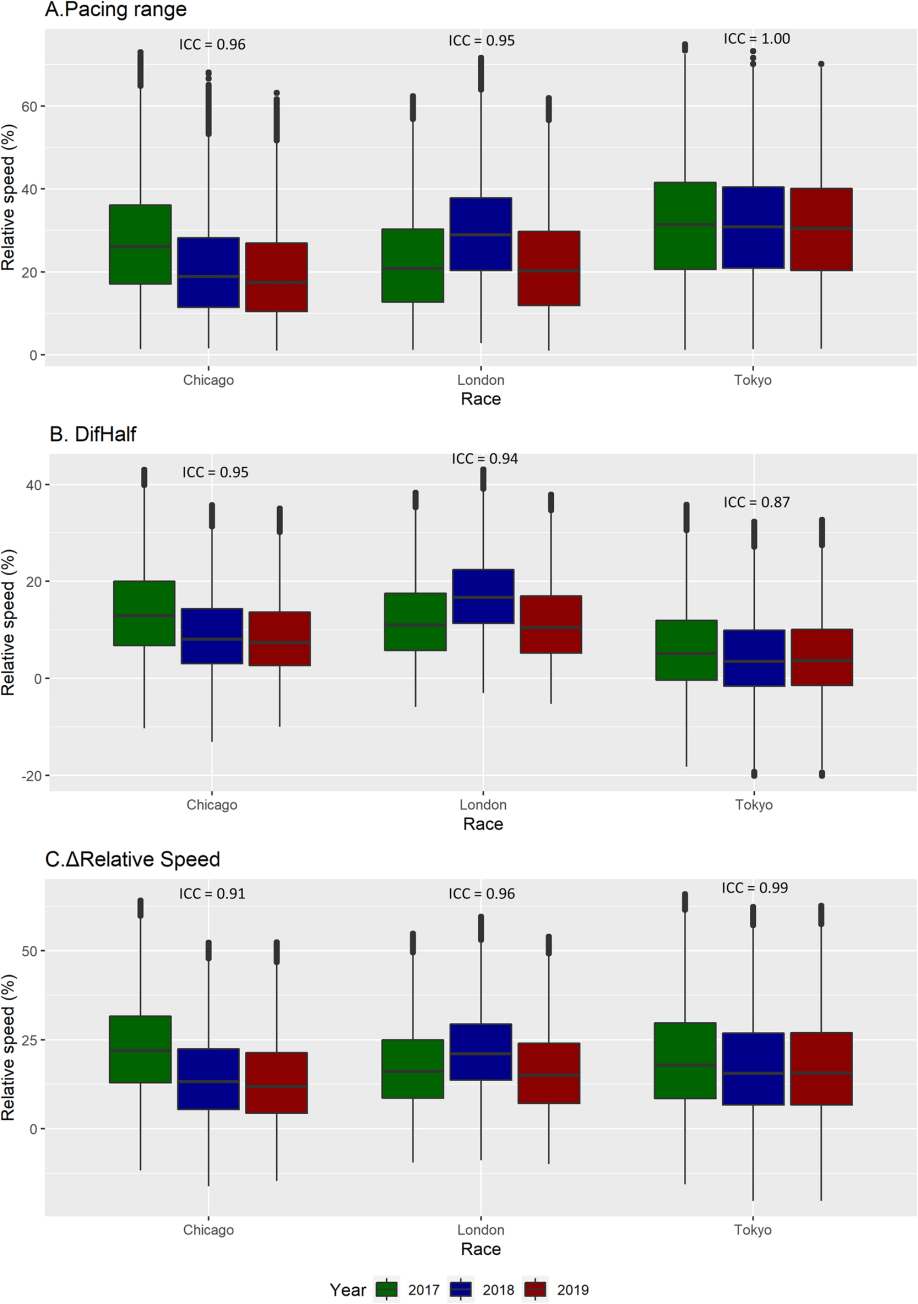
Boxplots of pacing range (**A**), DifHalf (**B**), and ΔRelative speed (**C**) of the marathons assessed. Intraclass correlation coefficients (ICC) between editions are shown for each marathon. No differences (*p* > 0.05 and ES < 0.8) were observed between marathon editions.

Moreover, pacing variables showed excellent ICC coefficients between editions (Fig. 4; ICC > 0.87) and did not present any differences between marathon editions (*p* > 0.05 and ES < 0.8).

**Figure 5:**
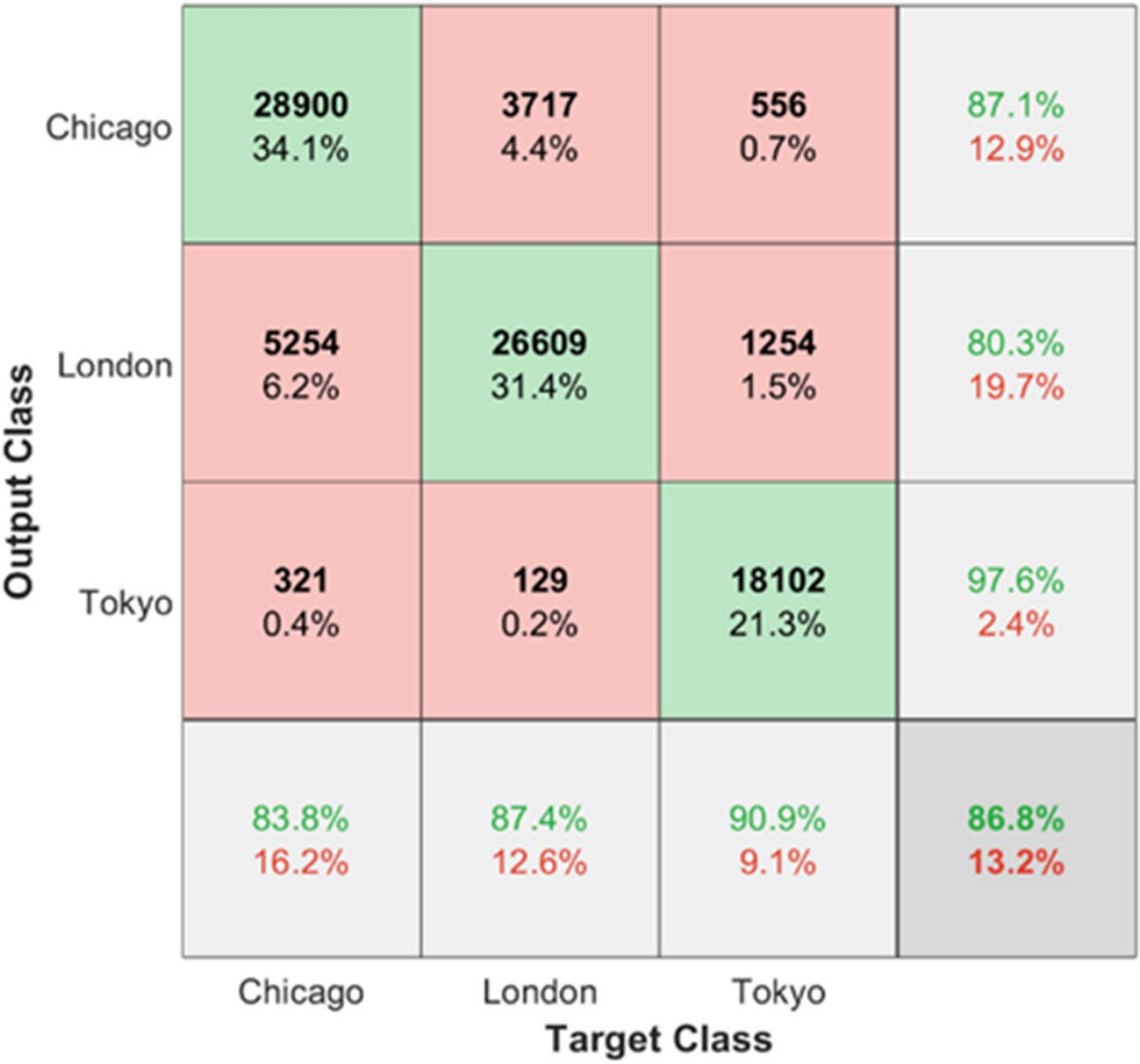
Matrix Confusion of the test dataset.

The ANN structure reached 86.8% of total accuracy. Detailed sensitivity and specificity values for each marathon are demonstrated using a confusion matrix (Fig. 5), showing that most false positives and false negatives were lower than the 7% of the cases, and 87, 80, and 98% of runners were correctly classified for Chicago, London and Tokyo races, respectively.

## Discussion

The aim of this study was to analyze pacing profile consistency in three consecutive editions of three different marathon competitions. The main results were that, although the ICC coefficients of relative speed at the different pacing sections, taking runner time categories into account, were excellent, some differences were observed between editions for some sections and categories of the Chicago and London marathons. The 2018 Chicago edition presented higher relative speed at km 21.1 and lower relative speed at the end of the race for sub-2:30 runners, and lower relative speed at the end of the race for sub-4:00 runners. 2018 London edition presented higher relative speed in the first 21 km than the other two editions for runners from sub-2:30 to sub-4:00. Pacing variables also presented excellent ICC coefficients with no differences between editions. Finally, artificial intelligence showed an accuracy of the 86.8% to classify the data of the runners in three marathons, suggesting a consistency between editions with identifiable differences between races.

Coaches are starting to analyze pacing profile of a specific race to plan their runner’s competition. Similarly, for race organizers, to analyze this pacing profile would enable to take decisions on the organization. However, to know if this analysis is valid, it is necessary to assess if the races are consistent in the different editions. To our knowledge, this is the first study analyzing consistency in pacing according time categories of different marathons editions. Our results shows that marathon editions presented excellent consistency, understanding this interpretation based on the excellent ICC values of relative speed at the different pacing sections, also excellent ICC values of pacing variables that can define the pacing profile of the race, and also by the good accuracy observed by the artificial intelligence model assessed. This result has an important practical application as it confirms that analyzing the pacing profile of a specific race can be useful for different purposes. Firstly, it is of use to runners and coaches aiming to plan the pacing strategy of the race according mostly to their performance level demands. Negative and even pacing throughout a race has been suggested as the most successful profile in a marathon^2,4,6,8,15,21^. However, it has also been observed that positive pacing can be a good strategy to achieve the best performance in specific races with particular course profile^33^. The fact that the pacing profile of the race is consistent means that a runner can analyze pacing profile of previous editions of a specific race and take into account in a runner's strategy^8^, cause it seems a consequence of performance probably due to different interaction of factors limiting performance such as psychological, physiological, metabolic, and cardiovascular demands^2,11,19,20,27^. Secondly, it is useful for organizers when it comes to evaluating how organizational changes in the edition could alter the pacing profile of the competition, if their goal is to facilitate runners records. An example of this is how most races try to reduce crowding at the beginning of the race due to its effect on the relative speed of the initial section^34^. The pacing variables' ICC coefficients were also excellent, suggesting that these variables could be used to characterize a specific marathon. Each one of the three variables assessed provides different information about the pacing characteristics of the race. The DifHalf is related to the facility of performing one of the types of profile (positive or negative pacing profile) which is of interest to coaches and elite athletes for improving marks or breaking marathon records^6,15^. Moreover, the pacing range is related to variability and ΔRelative speed to compare the beginning and the end of the race^8^.

ICC coefficients between the three marathons were also assessed to understand the interaction between category and race profile. Unsurprisingly, the ICCs were lower when evaluating the three competitions together. However, it was observed that these coefficients can still be considered very good (ICC > 0.75). This is in agreement with the results of Oficial-Casado and colleagues^8^, who observed that, although a specific marathon and its characteristics affect pacing, the effect of time category is decisive. Faster runners have a much more homogeneous profile throughout the race, and, as the time category increases, these runners perform the first half faster to the detriment of the second half of the race (positive pacing)^2,8,21^.

Concerning the differences observed between editions in certain categories and sections, we can just speculate on the reason for the results obtained due to the type of study carried out. In the case of the Chicago Marathon, the differences observed are in two categories (sub-2: 30 and sub-4:00) and concrete sections (km 20 and 40). We could then hypothesize that these differences are due to collective behaviors^27^ and specific runners of the 2018 edition. However, London presents more obvious differences between 2018 and the other two editions, and that is also found in most categories. The information available on the marathon website shows that the same route was taken in the three editions, and no logistical changes were found so that this reason can be ruled out. However, one reason that can explain these differences is the higher temperature observed in the 2018 edition. This higher environmental temperature could explain the faster relative speed in the first sections of the race^35,36^, and also a decrease in speed in the second half of the race due to thermal stress^35,37^. Further studies could explore the effect of environmental conditions on pacing in a controlled laboratory study.

Finally, the artificial intelligence model was able to identify the race with an accuracy of 87% based only on pacing profile data. This result provides confidence that the profile is specific to each race, and it is also consistent across different editions, although it may present some differences.

The main limitation of this study is the fact that reproducibility was not assessed in controlled laboratory conditions, which would allow us to control more factors that could affect pacing. Clearly, however, that kind of laboratory study would also have several limitations such as low ecological validity and small sample size, in contrast to the positive aspects of the current study that analyzed 282,808 runners' data with robust statistical results. We assume that these results are generalizable for gold label marathons with no changes in their route. However, consistency could be different for non-gold label marathons, or with small modifications in the organization, route or with lower number of participants, which encourages further research. Finally, as the data was obtained anonymously, factors such as the age or the sex were not analyzed.

In conclusion, although some differences were found between editions in certain sections and runners’ time categories of the three different marathons, the data suggested that an excellent consistency of the pacing profile was observed between their editions. Therefore, the study of the pacing profile for a specific marathon can be helpful to runners, coaches and marathon organizers for planning the race and improving its organization.

## Data Availability

The datasets generated and analysed during the current study are available in the Mendeley repository (doi: 10.17632/xvfvk2zvhw.1; https://data.mendeley.com/datasets/xvfvk2zvhw/1).
